# Methylcap-Seq Reveals Novel DNA Methylation Markers for the Diagnosis and Recurrence Prediction of Bladder Cancer in a Chinese Population

**DOI:** 10.1371/journal.pone.0035175

**Published:** 2012-04-17

**Authors:** Yangxing Zhao, Shicheng Guo, Jinfeng Sun, Zhaohui Huang, Tongyu Zhu, Hongyu Zhang, Jun Gu, Yinghua He, Wei Wang, Kelong Ma, Jina Wang, Jian Yu

**Affiliations:** 1 State Key Laboratory of Oncogenes and Related Genes, Shanghai Cancer Institute, Renji Hospital, Shanghai Jiao Tong University School of Medicine, Shanghai, China; 2 Ministry of Education's Key Laboratory of Contemporary Anthropology and State Key Laboratory of Genetic Engineering, School of Life Sciences and Institutes of Biomedical Sciences, Fudan University, Shanghai, China; 3 Oncology Institute of Wuxi, The Fourth Affiliated Hospital of Suzhou University, Wuxi, China; 4 Department of Urology, Shanghai Key Laboratory of Organ Transplantation, Zhongshan Hospital, Fudan University, Shanghai, China; Cleveland Clinic Foundation, United States of America

## Abstract

**Purpose:**

There is a need to supplement or supplant the conventional diagnostic tools, namely, cystoscopy and B-type ultrasound, for bladder cancer (BC). We aimed to identify novel DNA methylation markers for BC through genome-wide profiling of BC cell lines and subsequent methylation-specific PCR (MSP) screening of clinical urine samples.

**Experimental Design:**

The methyl-DNA binding domain (MBD) capture technique, methylCap/seq, was performed to screen for specific hypermethylated CpG islands in two BC cell lines (5637 and T24). The top one hundred hypermethylated targets were sequentially screened by MSP in urine samples to gradually narrow the target number and optimize the composition of the diagnostic panel. The diagnostic performance of the obtained panel was evaluated in different clinical scenarios.

**Results:**

A total of 1,627 hypermethylated promoter targets in the BC cell lines was identified by Illumina sequencing. The top 104 hypermethylated targets were reduced to eight genes (VAX1, KCNV1, ECEL1, TMEM26, TAL1, PROX1, SLC6A20, and LMX1A) after the urine DNA screening in a small sample size of 8 normal control and 18 BC subjects. Validation in an independent sample of 212 BC patients enabled the optimization of five methylation targets, including VAX1, KCNV1, TAL1, PPOX1, and CFTR, which was obtained in our previous study, for BC diagnosis with a sensitivity and specificity of 88.68% and 87.25%, respectively. In addition, the methylation of VAX1 and LMX1A was found to be associated with BC recurrence.

**Conclusions:**

We identified a promising diagnostic marker panel for early non-invasive detection and subsequent BC surveillance.

## Introduction

Bladder cancer (BC) is one of the leading causes of cancer-related morbidity and mortality and the sixth most common cancer in the world [1]. In China, the incidence of BC continues to rise [2]. BC incidence increases with age; the average age at the time of diagnosis is approximately 60 years, and it is 3 times more common in men than in women [3]. Smoking and exposure to carcinogens have been identified as risk factors [4]. Approximately 75–80% of new BC cases occur as superficial or carcinoma *in situ* lesions, whereas the remaining 20–25% present as a more advanced disease, with a poor prognosis. However, even in the superficial tumors, only 20% are curable. Approximately 60–70% of patients will relapse within 5 years, and 10–20% of tumors will progress to a more aggressive disease [5], which necessitates frequent monitoring for disease recurrence [6]. Cystoscopy is the most common diagnostic BC procedure, and it shows high sensitivity (SN) and specificity (SP). However, cystoscopy requires high operator proficiency, and the invasive nature of cystoscopy reduces its value as a screening tool. Other optimal methods are needed for the early, non-invasive detection and surveillance of BC.

**Table 1:** Summary of the clinical-pathological data of urine samples from bladder cancer patients and controls.

	Bladder cancer	Normal control	Nontumor urinary lesions	Surgery resect	Clinical cystoscope
	n = 212	n = 149	n = 41	n = 21	n = 48
Gender					
F	46	71	16	3	14
M	166	78	25	17	34
Age					
–30	1	8	1	0	0
31–40	1	14	4	1	2
41–50	27	29	8	4	1
51–60	41	23	6	2	4
61–70	44	26	9	4	6
71–	98	49	13	10	35
Range	29–91	22–90	16–89	35–88	31–90
Median	69	61	61	69	72
Mean±SD	66.85±12.74	59.77±17.14	60.24±17.02	65.33±14.44	68.85±13.44
Grade					
I	75				
II	120				
III	25				
Stage					
Oa	3				
I	134				
II	63				
III	7				
IV	5				
Relapse					
Primary	157				
Recurrency	55				
Cystitis			17		
urinary tract infection			13		
kidney stones			5		
prostatitis			3		
Nephritis & nephroticsyndrome			3		

notes: Table 1 does not contain the information of the samples used for small cohort screening.

The epigenetic facet of the genome connects the genotype of an individual to environmental influences that shape the heritable gene transcription pattern and therefore influence the phenotype of the cell [7]. Regulation at the epigenetic level is critical for the development of higher eukaryotes [8], and aberrant regulation can directly and/or indirectly influence the genetic integrity and gene expression pattern of cells, resulting in the development of various types of disorders, including cancer [9]. The local hypermethylation of tumor-suppressor genes [10] and the global hypomethylation of genomic DNA [11], [12], [13] often occur in human cancers [14], [15]. Recent advances have shown that the abnormal hypermethylation of tumor-suppressor genes is an emerging biomarker for cancer diagnosis and prognosis. In BC, several methylated genes have been identified, and their role as urinary markers has also been evaluated [16]. These studies have clearly demonstrated the advantages of using multiple gene hypermethylation analyses in tissue and urine samples to obtain diagnostic and prognostic information. For example, in our previous study, we identified a panel of 11 methylated genes that could be used as urine-based markers for BC screening; however, this panel had limitations. The gene number in the panel was too large for convenient clinical testing, and the specificity was insufficient [17]. To improve the efficiency of diagnosis and identify gene targets that can predict recurrence or progression, it is necessary to find novel targets and validate their clinical value in a large cohort. However, thus far, few BC studies have used a genomic-scale high-throughput approach to screen for differentially methylated genes [18], [19]

MethylCap-seq is a recently developed technique for the genome-wide profiling of DNA methylation; this technique consists of capturing the methylated DNA fragments by their methyl-CpG binding domains (MBDs) and the subsequent deep sequencing of eluted DNA. A salt-gradient elution classifies the genome into fractions with different methylation states. The profiles obtained in this way provide a detailed genome-wide map of methylated regions and allow the detection of DNA methylation in different genomic regions [20].

**Figure 1:**
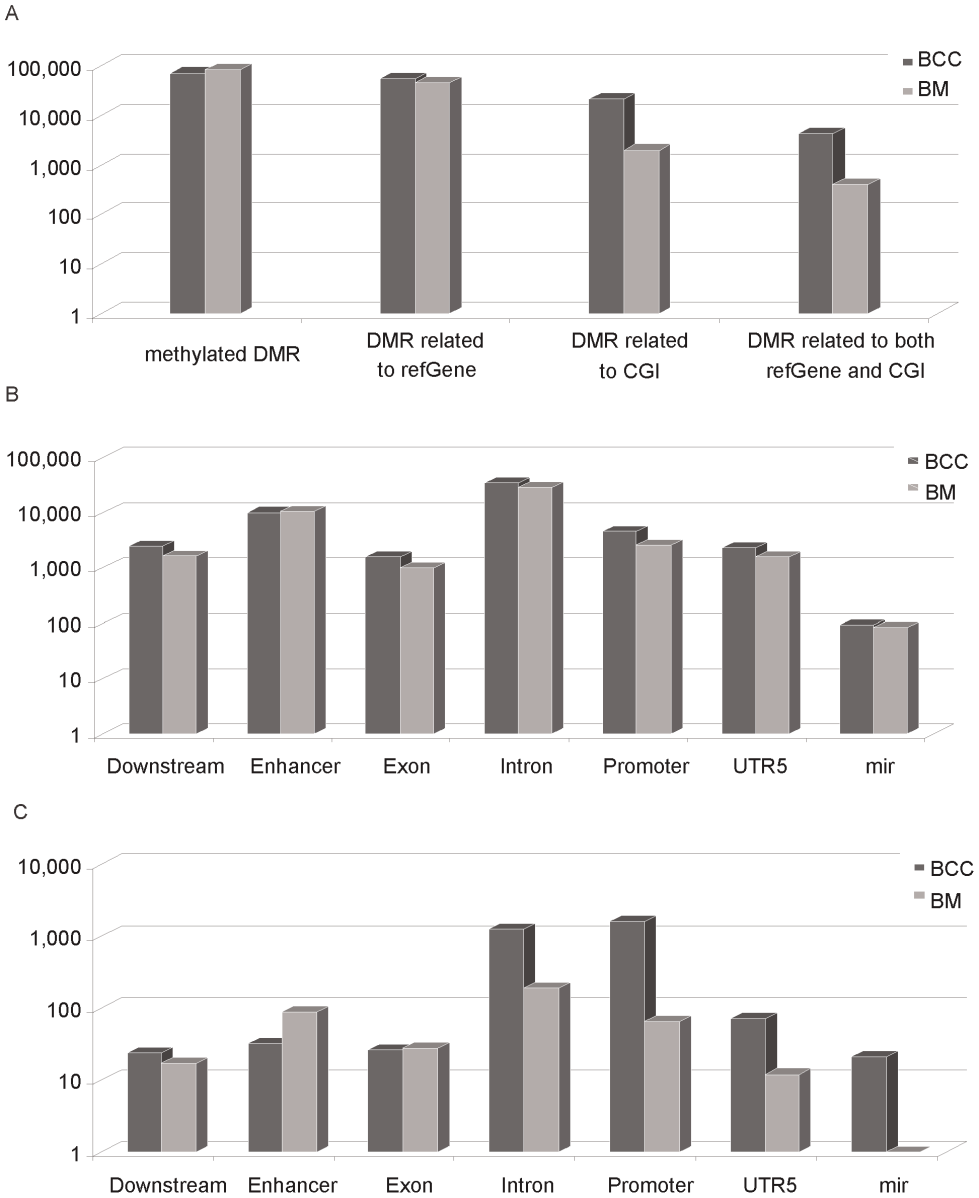
General characteristics of BCC-specific DNA methylation patterns determined by MBD methylCap-seq. The genomic context is defined as those found in the UCSC database. The featured differential methylation region (DMR) distribution in BCCs and BMs: (A) general distribution; (B) distribution in refGene; (C) distribution in both refGene and CGI.

In this study, we first employed MBD MethylCap-seq to obtain an overall methylation profile of BC cell lines (BCCs), which we believed would provide information regarding BC-specific aberrant methylation. Subsequently, the DNA from the urine of BC patients was screened for the top 100 hypermethylated targets from BCCs to identify BC-specific methylation sites. This number gradually decreased, and the marker quality improved during the screening process steps. Finally, we acquired a novel set of diagnostic DNA methylation markers that may be used for the early, non-invasive detection and surveillance of BC.

## Materials and Methods

### Patient and control sample collection

With informed consent and the approval of Medical Institutional Review Board of Zhongshan Hospital, Fudan University, urine specimens were collected from 212 patients with a confirmed BC diagnosis, 41 patients with noncancerous urinary lesions hospitalized during the same time period, and 149 normal controls. A group of paired voided urine samples was also collected from the 21 BC patients before and after surgery that included transurethral resection of bladder cancer plus intravesical chemotherapy (TURBC+IC). In addition, another group of 48 urine samples was collected from patients strongly suspected of having a malignant bladder tumor. All the BC patients and controls came from two hospitals: the Urology Department at Zhongshan Hospital, Shanghai, China and the No. 2 Shimen Street Community Health Center, Jingan District, Shanghai. The samples were collected between 2006 and 2009. The tumor-node-metastasis (TNM) staging/classification of the BC patient samples was determined according to American Joint Committee on Cancer guidelines [21] (Table 1). The samples (50 mL of fresh urine) were centrifuged at 3,000 rpm for 10 minutes. The supernatant was then decanted, and the pellet was washed once with 1× phosphate buffer saline (PBS) and immediately frozen at −80°C.

**Figure 2:**
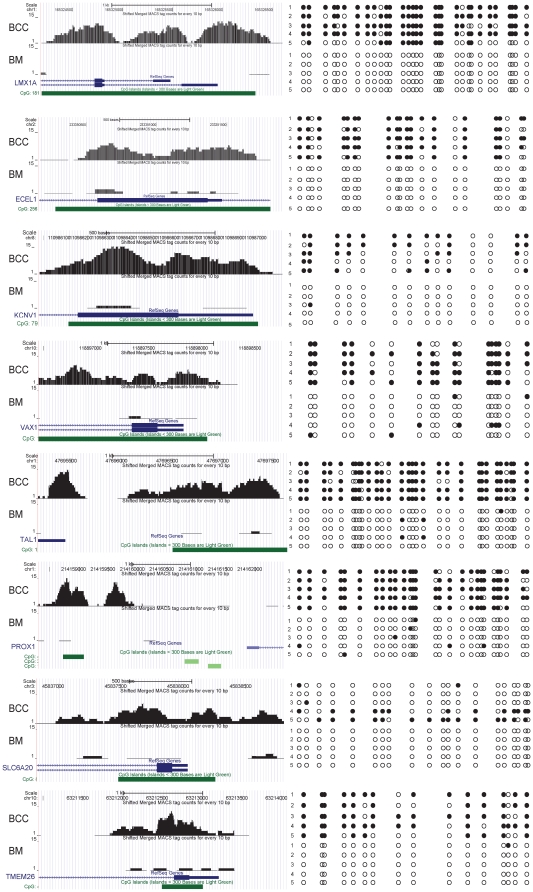
Representative BSP confirmation of the MBD methylCap/seq library. The Wig picture from the UCSC database is on the left, and the BSP result where at least 5 clones were sequenced for each locus is on the right. The black circle indicates methylated C in the CpG context; the white circle indicates unmethylated C in the CpG context.

### Cell lines and normal bladder mucosal tissue

Two BCCs, T24 (ATCC No: HTB-4) and 5637 (ATCC No: HTB-9), were purchased from the American Type Culture Collection (ATCC, Manassas, VA) and cultured until they reached the log phase in L-DMEM medium containing 10% fetal bovine serum (FBS) at 37°C in a 5% CO_2_ humidified incubator. The cells were harvested by scraping, and the cell pellets were rinsed twice with 1× PBS. Two normal bladder mucosal tissues (BM1 and BM2) were obtained from healthy organ donors. For economic reasons, the two BCCs were pooled to construct the BCC library, as were the two BM samples. In this way, we could acquire all of the methylation information from each of the 2 samples.

**Figure 3:**
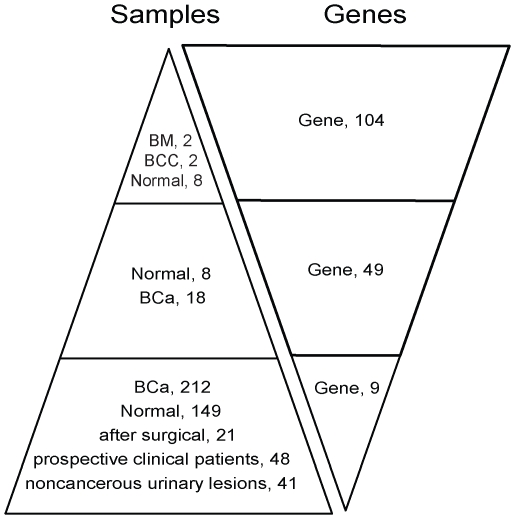
Flow chart describing the number of samples and candidate genes in the screening process. MBD methylCap-seq information was used to select genes differentially hypermethylated in bladder cancer. The sample number in the figure refers to that of the screening process. The methylation status of the target gene was screened in samples with different sizes. In this phase, the number of samples progressively increased, while the number of genes progressively decreased.

### MBD-methylCap-sequencing

A DNA preparation from frozen tissues and cell lines was generated using a conventional proteinase K/organic extraction method as previously described [22]. Equal amounts of DNA from the 5637 and T24 cells were combined to form the BCC library, and equal amounts of DNA from the BM1 and BM2 cells were combined to form the BM library. In 1.5-mL centrifuge tubes, 1.5 µg of the combined DNA samples (BCC or BM) in 100 µl TE buffer were sonicated to yield the desired size range (200–300 bp). End-repair, adenosine base addition and adaptor ligation steps were performed as previously described [23].

The commercial MethylMiner™ Methylated DNA Enrichment Kit (Invitrogen Inc., Carlsbad, CA, USA) was used to select methylated DNA for sequencing. For each group, 1.2 µg of the treated DNA was processed according to the manufacturer's protocol. After a NaCl gradient elution, we collected the final two fractions of highly methylated DNA, which corresponded to 1 M and 2 M NaCl concentrations. The spike-in DNA control supplied by the kit was used to confirm the accuracy of the assay. The recovered DNA (in the nanogram range) was quantified by Qubit™ (Invitrogen), and 12 cycles of PCR amplification were performed to obtain enough material (in the microgram range) for deep sequencing. Finally, 1 µg of the PCR product was applied to the Genome Analyzer II (Illumina, Inc., San Diego, CA) to generate 75 bp-long unpaired reads. PCR duplicates were removed from the analysis. We used BWA alignment tools with the default settings to map these reads to the hg19 human genome reference assembly (UCSC) [24]. Next, the peaks (hypermethylated regions) were identified using MACS software [25], and the human CpG islands (CGIs) were downloaded from the UCSC database. The genomic methylation profile generated was uploaded to a public database (Gene Expression Omnibus: GSE 33839)

**Table 2:** The diagnosis performance of the 9 studied methylation targets, individually or in panel, in bladder cancer versus normal or nontumor urinary lesion controls.

	Bladder cancer	Bladder cancer vs Normal control (n = 149)					Bladder cancer vs Nontumor urinary lesions (n = 41)				
	Sensitivity(%)	Specificity (%)	AUC(95% CI)	PPV	NPV	*P*	Specificity (%)	AUC(95% CI)	PPV	NPV	*P*
	(pos./total)	(neg./total)		(%)	(%)		(neg./total)		(%)	(%)	
*VAX1*	42.45(90/212)	95.31(142/149)	73.3(68.0–78.6)	92.78	53.79	<0.0001	87.81(36/41)	59.5(52.5–66.5)	94.74	21.43	0.0002
*KCNV1*	36.92(84/212)	93.96(140/149)	71.3(65.7–76.8)	90.32	52.24	<0.0001	95.12(39/41)	60.5(53.6–67.5)	97.67	23.35	<0.0001
*ECEL1*	26.89(57/212)	97.31(145/149)	70.8(64.7–76.8)	96.55	48.33	<0.0001	97.56(40/41)	59.3(51.7–67.0)	98.28	20.51	0.0002
*TMEM26*	26.42 (56/212)	96.64(144/149)	69.4(62.9–75.8)	91.80	48.00	<0.0001	97.56(40/41)	60.3(52.5–68.0)	98.25	20.41	<0.0001
*PROX1*	24.53(52/212)	98.66(147/149)	71.1(65.0–77.3)	96.30	47.88	<0.0001	100.0(41/41)	59.1(61.2–66.9)	100.0	20.40	<0.0001
*TAL1*	24.83(52/212)	98.66(147/149)	72.5(66.8–78.3)	93.44	48.51	<0.0001	100.0(41/41)	60.4(52.9–68.0)	100.0	20.40	<0.0001
*SLC6A20*	15.57(33/212)	97.89(146/149)	69.7(62.3–77.0)	91.67	44.92	<0.0001	100.0(41/41)	59.3(50.2–68.4)	100.0	18.64	0.0039
*LMX1*	9.43(20/212)	98.66(147/149)	67.1(57.5–76.8)	90.91	43.36	<0.0001	100.0(41/41)	58.8(47.5–70.1)	100.0	17.60	0.0404
**Panel 1**	76.89(163/212)	88.59(132/149)	82.8(78.5–87.1)	90.56	72.93	<0.0001	85.36(35/41)	84.3(79.1–89.5)	96.45	41.67	<0.0001
*CFTR*	52.35(111/212)	96.64(144/149)	77.4(72.6–82.1)	95.69	58.78	<0.0001	97.56(40/41)	63.8(57.1–7.02)	99.11	28.37	<0.0001
**Panel 2**	88.68(188/212)	87.25(130/149)	89.9(86.7–93.2)	90.82	84.42	<0.0001	85.36(35/41)	90.0(85.9–94.2)	96.91	59.32	<0.0001

Panel 1: *VAX1,KCNV1,TAL1,PPOX1*.

Panel 2: *CFTR,VAX1,KCNV1,TAL1,PPOX1*.

### MSP and BSP

Bisulfite conversion and PCR analysis were performed as previously described [22]. The bisulfite sequencing PCR (BSP) and methylation-specific PCR (MSP) primer pairs were designed with the assistance of appropriate online software (http://www.urogene.org/methprimer/index1.html; Table S1, Table S2). The MSP products were cloned and verified by sequencing. The *in vitro* methylated DNA from the 5637 and T24 cells was obtained with the CpG methyltransferase M. Sss I (NEB) and used as a positive control. Water was used as a no-template control. Bisulfite sequencing was performed as previously described [17], and the PCR amplicons were gel-purified and cloned into a pBS-T II vector (TianGen Incl., Beijing, China). At least 5 clones were individually sequenced to ascertain the methylation patterns of the targeted locus. The BSP methylation percentage was calculated as the number of methylated cytosines divided by the total number of cytosines in all of the amplicons analyzed.

### Statistics

The major statistical endpoints in this study involved comparing the methylation statuses of genes thought to be associated with BC and their relevant clinical variables in the control and cancer patients. The presence or absence of methylation using MSP was evaluated to determine the associations between methylation status and cancer or its clinical variables using cross-tabulations and the appropriate χ^2^ or Fisher's exact t-tests. The association of BC recurrence with gene methylation and clinical variables was assessed by means of uni- and multivariate logistic-regression analyses. The outcome selected for the follow-up analysis was the cumulative hazard of recurrence, which was defined as the time from BC diagnosis to the date of tumor recurrence. Uni- and multivariate Cox proportional hazards models were used to assess the effects of gene methylation and other clinical variables of disease recurrence. The cumulative recurrence hazard curve was generated by the Kaplan-Meier method and verified by the log-rank test. All the statistical calculations were performed using the SPSS 13.0 software statistical package (SPSS Inc., Chicago, IL). Two-sided P values less than 0.05 were considered significant.

**Figure 4:**
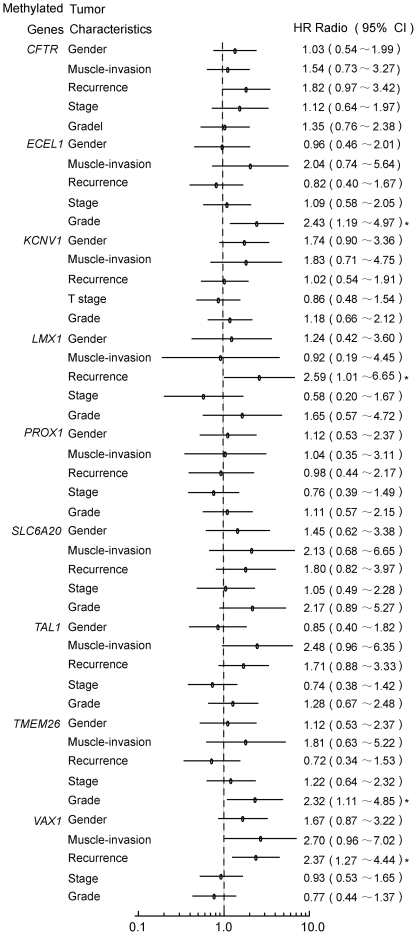
The relationship between gene methylation (hazard ratio) and BC tumor characteristics. A multiple univariate logistic regression was performed with the use of methylation data of the 9 genes to evaluate the relationship between gene methylation and the clinicopathological characteristics of BC. The details are described in the text. *P<0.05.

**Table 3:** The multivariate hazard ratios and 95% confidence intervals for the estimated risk of recurrence among recurrent cases compared with primary cases according to LMX1A and VAX1 methylation (separated or combined) and clinical variables.

Clinical variables		Number of case	Number of recurrence	(%)	HR	95% CI	*P*
Overall		212	55	25.94%			
***LMX1A*** Methylation	Negative	192	46	23.96%			
	Positive	20	9	45.00%	2.63	(1.01∼6.85)	0.012
Gender	Female	46	15	32.61%			
	Male	166	40	24.10%	0.69	(0.33∼1.42)	0.555
Muscle invasion	Absent	178	44	32.35%			
	Present	34	11	24.72%	1.76	(0.71∼4.37)	0.217
Stage	0a+I	137	36	26.28%			
	II+III	75	19	25.33%	1.00	(0.46∼2.16)	0.920
Grade	I	73	23	31.51%			
	II+III	139	32	23.02%	0.59	(0.29∼1.18)	0.171
***VAX1*** Methylation	Negative	122	23	18.85%			
	Positive	90	32	35.56%	2.27	(1.20∼4.32)	0.047
Gender	Female	46	15	32.61%			
	Male	166	40	24.10%	0.80	(0.38∼1.68)	0.313
Muscle invasion	Present	178	44	24.72%			
	Absent	34	11	32.35%	1.79	(0.71∼4.49)	0.226
Stage	0a+I	137	36	26.28%			
	II+III	75	19	25.33%	0.96	(0.45∼2.08)	0.998
Grade	I	73	23	31.51%			
	II+III	139	32	23.02%	0.61	(0.30∼1.24)	0.134
***LMX1A*** &***VAX1***	Negative	200	48	24.00%			
	Positive	12	7	58.33%	4.73	(1.39∼16.08)	0.013

Hazard Ratio are reported on the basis of the multivariate logistic-regression model.

## Results

### Genomic methylation profiling of the BCC and BM libraries revealed characteristic methylation patterns in BC

We profiled the genome-wide DNA methylation status of the BCC and BM libraries by generating MBD-methylCap-enriched DNA libraries. The MBD-enriched fractions were subjected to high-throughput sequencing using an Illumina Genome Analyzer II to obtain comprehensive methylation maps. Conceptually, the hypermethylated DNA fragments were enriched during the library construction process; therefore, the acquired sequencing reads correspond with the hypermethylated regions on the genome. Deep sequencing of the prepared BCC and BM libraries produced approximately 6 million reads (75 bases/read) for each library, which were derived from approximately 470 million bases (Table S3). This amount of sequencing bases could cover the genomic CGI in approximately 10 times the depth. Thus, the data sets successfully provided genome-wide information.

**Table 4:** The univariate and multivariate analyses of the recurrence prognostic values of various factors according to VAX1 and LMX1A methylation (separated or combined) and clinical variables.

Clinical variables		Number of case	Number of recurrence	(%)	Cox-ranked univariate			Cox-ranked multivariate		
					HR	95% CI	*P*	HR	95% CI	*P*
Overall		145	37	25.5%						
***VAX1***	Negative	93	18	19.4%						
	Positive	52	19	36.5%	1.92	1.00∼3.65	0.048	2.11	(1.08∼4.11)	0.029
Gender	Female	29	7	24.1%						
	Male	116	30	25.9%	0.86	0.38∼1.95	0.711	0.72	(0.31∼1.67)	0.440
Muscle invasion	Absent	128	33	25.8%						
	Present	17	4	23.5%	1.05	0.37∼2.96	0.928	0.93	(0.30∼2.85)	0.893
Treatment	TURBT+IC	78	16	20.5%						
	PC	67	21	31.3%	0.93	0.48∼1.79	0.822	0.84	(0.42∼1.67)	0.614
Stage	0a+I	97	26	26.8%						
	II+III	48	11	22.9%	1.14	0.56∼2.30	0.721	1.30	(0.61∼2.77)	0.500
Grade	I	46	10	21.7%						
	II+III	99	27	27.3%	0.77	0.37∼1.60	0.488	0.66	(0.30∼1.43)	0.290
***LMX1a***	Negative	136	32	23.5%						
	Positive	9	5	55.6%	3.08	1.20∼7.95	0.019	3.31	(1.27∼8.59)	0.014
Gender	Female	29	7	24.1%						
	Male	116	30	25.9%	0.86	0.38∼1.95	0.711	0.83	(0.36∼1.89)	0.649
Muscle invasion	Absent	128	33	25.8%						
	Present	17	4	23.5%	1.05	0.37∼2.96	0.928	1.09	(0.35∼3.36)	0.881
Treatment	TURBT+IC	78	16	20.5%						
	PC	67	21	31.3%	0.93	0.48∼1.79	0.822	0.88	(0.45∼1.75)	0.724
Stage	0a+I	97	26	26.8%						
	II+III	48	11	22.9%	1.14	0.56∼2.30	0.721	1.28	(0.59∼2.75)	0.529
Grade	I	46	10	21.7%						
	II+III	99	27	27.3%	0.77	0.37∼1.60	0.488	0.67	(0.31∼1.46)	0.312
***LMX1a*** & **VAX1**	Negative	140	33	23.6%						
	Positive	5	4	80.0%	6.40	2.24∼18.29	0.001	7.25	(2.41∼21.79)	0.014

Hazard ratios, 95% confidence intervals, and their conrresponding P-values were calculated using univariate and multivariate Cox proportional hazard medels.

When these reads were mapped into the genome, the uneven distribution formed peaks that represent the hypermethylated regions of the genome. In total, we obtained 210,051 peaks (mean length of peaks: 778 bp) in the BCCs and 229,538 peaks (mean length of peaks: 659 bp) in the BMs (*P*<0.001, MACS2.0; Table S3).

To obtain the relative methylation information, we compared the peaks between the BCCs and the BMs. Nearly two-thirds of the total peaks were common between the two libraries, and we ignored them for this analysis. The remaining one-third of the peaks were unique to either the BCCs or the BMs, which we termed the differentially-methylated regions (DMRs) that represent the relatively high methylation status of the genomic regions compared with its counterpart. We obtained 70,432 and 83,690 DMRs in the BCCs and the BMs, respectively (Table S3, Figure 1 A). This large amount of DMR was scattered within different genomic contexts, and we analyzed the association of the DMR with the different genomic contexts. The refGene-related DMR was 55,237 and 45,522 in the BCCs and the BMs, respectively, indicating a roughly balanced distribution in both libraries (Table S3, Figure 1 B). However, when DMRs within the CGIs were investigated, we found that the BCCs retained 21,179 DMRs, while the BMs retained only 1,945; this represents a ten-fold difference. When the DMRs that occurred within the CGIs of the refGene were studied, the BCCs and the BMs contained 4,256 and 201 DMRs for each library, respectively. Lastly, when promoter involvement was calculated, 1,627 and 66 DMRs were associated in this region in the BCCs and the BMs, respectively (Table S3, Figure 1 C). Taken together, aberrant hypermethylation occurred more frequently in the CGIs and the promoter regions of the BCCs. The following studies of the methylation marker selection focused on BCC promoters.

### Validation of the distinct BCC methylation profile using bisulfite sequencing

To confirm that the library accurately reflected the real methylation status of the studied material, we selected 24 different hypermethylated targets for bisulfite sequencing verification. Of these, 22 targets were scattered within the 1,627 promoter-related DMRs in the BCCs, including 17 from the top 100 targets and 5 from the 100 to 1,627 range; the other 2 targets were from the 66 DMRs in the BMs. Encouragingly, among the 24 targets being verified, the BSP results of 23 genes were highly consistent with the methylation information acquired in the library (a representative result is shown in Figure 2, Table S4), suggesting that the BCC and BM libraries were highly reliable for methylation information. To assess the potential of the library for suggesting clinical diagnostic targets, we searched the methylation information of targets identified in our previous work in these two libraries [17]. A total of 90% (19/21) of those targets were hypermethylated in the BCC library (data not shown). In addition, we also investigated the BC-specific markers reported by others [19], [26]. Eight of the 9 targets were located in our BCC library as hypermethylation loci. Taken together, the present BCC and BM libraries provided sound hypermethylation information with respect to BC status.

### MSP screening on a small cohort of urine samples for potential biomarkers generated 8 gene targets

Presented with the large aberrant methylation information provided by methyCap-seq, we needed a method to filter the BCC methylation results to identify feasible BC markers. We adopted the strategy of beginning the screening process with a large number of targets in a few samples and gradually reducing the targets with an increase in samples (Figure 3). When subjected to the MSP limitations, only the top 104 of the 1,627 hypermethylated promoters of the BCCs were screened in the urine DNA samples of 8 normal controls (BNs); the same BCC and BM samples used in the MethylCap-seq portion of the study were included as controls. In these conditions, only the targets that showed methylation in at least one of the two BCCs but not in more than 2 of the 8 BN proceeded to the next screening (a representative MSP result is shown in Figure S1). Because they did not meet these criteria, 55 targets were removed from the first round of screening. Forty-nine targets proceeded to the second round of urine DNA screening from an additional 8 BNs and 18 BC patients. We selected the targets that showed methylation in at least 3 of the 18 BC samples but 0 or 1 of the 8 BN samples. In this stage, 8 genes (VAX1, KCNV1, ECEL1, TMEM26, TAL1, PROX1, SLC6A20, and LMX) met the conditions and were selected for their potential to discriminate BC from BN (Figure 3).

### Assessment of the diagnostic value of the 8 targets in a large cohort

To ensure that the potential of the BC candidate targets was reliably evaluated, we screened the 8 candidates in an independent test cohort with a large sample size. We obtained urine samples from a large cohort (n = 402) that included 212 BC patients, 149 normal controls, and 41 patients with noncancerous urinary lesions.

The methylation frequency of the 8 novel genes in the urine DNA from BC patients (212 cases) ranged from 9.43% to 42.45%, whereas the methylation frequency in normal controls (149 cases) ranged from 1.34% to 6.04%. All 8 targets showed a significant difference between the tumors and normal controls (*P*<0.0001), which favorably argued for their potential use as diagnostic markers (Table 2).

To differentiate tumor-specific methylation from possible methylation associated with benign disease, 41 noncancerous urinary lesions were included in our study (Tables 1 and 2). The methylation frequency of the 8 genes in this patient group ranged from 0.00% to 12.19%, supporting the notion that the origin of the hypermethylation was more closely linked to tumors than benign disease (*P*<0.04; Table 2). Therefore, these 8 targets may potentially serve as markers for the clinical detection of BC and to distinguish BC from normal controls and benign urinary lesions.

Given the heterogeneous nature of tumor methylation, a single methylated marker cannot provide adequate SN and SP for tumor diagnosis [27]. Therefore, combining a group of genes as a panel was an alternative option [28]. Taking the area under the curve (AUC) as a judgment of diagnostic ability, a combination of 4 genes (VAX1, KCNV1, TAL1, PROX1) was selected to form a diagnostic panel, which showed an SN of 76.89% and SP of 88.59% (Table S5).

To further improve the diagnostic potential of this panel, we added CFTR and SALL3, the two top BC targets from our previous work [17], which were also located within the 1,627 promoters related to DMRs in the BCCs. The CFTR exhibited decent potential for diagnosing BC, with an SN and SP of 52.36% and 96.64%, respectively (Table 2). However, SALL3 did not present a good SP in a large-cohort evaluation and was therefore removed (data not shown). Finally, a 5-gene panel (VAX1, KCNV1, TAL1, PROX1 and CFTR) was adopted that revealed diagnostic efficiency, with SN, SP, positive predictive value (PPV), and negative predictive value (NPV) of 88.68%, 87.25%, 90.82%, and 84.42%, respectively (Table 2). The similar results was also obtaind in a independent small cohort validation analysis consisted of 24 BC and 22 controls (data not shown).

### The diagnostic ability of the five-gene panel is comparable to cystoscopy

The performance of the five targets in the evaluation of suspected clinical patients is critical. Therefore, we used MSP to assess the urine of patients who had suspected uroepithelial malignancies. Of the 48 patients, 32 BC patients were eventually confirmed by cystoscopy, and 25 of these were MSP-positive for at least one of the five genes. Of the 16 patients who did not show malignancy by cystoscopy, 14 were negative for MSP. Therefore, the 5-gene target showed good conformity with cystoscopy (81.25%; 25 positive and 14 negative in a total of 48 patients by both procedures).

### The five-gene panel could also predict the effectiveness of surgical resection

All 21 (100.0%) of the BC patients were MSP-positive for at least 1 of the 5 genes before surgery, whereas only 2 of the 21 (9.5%) BC patients retained MSP-positive genetic loci after surgery (*P*<0.0001). These results add additional support to our previous discovery and hypothesis [17] and corroborate the idea that a close relationship exists between the methylation observed in urine sediment and the corresponding bladder tumor.

### The hypermethylation of VAX1 and LMX1A is important for predicting cancer recurrence

In addition to the relationship between the gene methylation status and the malignant phenotype of the tumor itself, we also studied the association between methylated targets and different clinical parameters.

**Figure 5:**
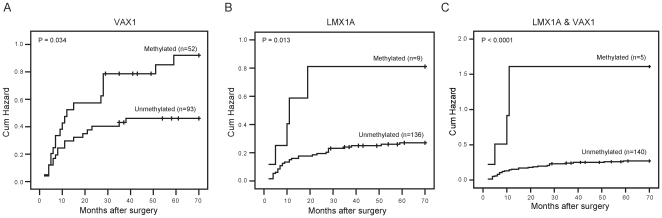
The role of urine DNA VAX1 methylation markers in the clinical prognosis of bladder cancer. Kaplan-Meier estimations of recurrence-free survival in the follow-up cohort of 145 patients according to the presence of methylated VAX1 and LMX1A (A and B). VAX1 or LMX1A methylation was significantly associated with poor prognosis for BC patients (P = 0.034 and P = 0.013, respectively). Analysis of two genes combined shows more statical power (P<0.0001).

The multiple univariate logistic-regression analysis of 9 gene targets (VAX1, KCNV1, ECEL1, TMEM26, TAL1, PROX1, SLC6A20, LMX1A and CFTR) showed that VAX1 and LMX1A methylation was more common in urine samples from recurrent cases than in samples from primary cases in the initial analysis of 212 BC patients (primary: 157; recurrence: 55), with HR = 2.37 (CI 95%, 1.27 to 4.44, P<0.05) and HR = 2.59 (CI 95%, 1.01 to 6.65, P<0.05), respectively (Figure 4). Multivariate logistic-regression models revealed that the HR of VAX1 and LMX1A were 2.27 (95%CI, 1.20 to 4.32; *P* = 0.047) and 2.63 (95% CI, 1.01 to 6.85; *P* = 0.012), respectively (Table 3). And the association was more evident when these two genes were analyzed in combination, with HR of 4.73(95%CI, 1.39 to 16.08; *P* = 0.013). The close association of these two genes with recurrence was evident in the follow-up data based on 145 cases (no recurrence: 108; recurrence: 37) with intact follow-up information. Multivariate Cox proportional hazard models revealed that the HR of VAX1 and LMX1A were 2.11 (95%CI, 1.08 to 4.11; *P* = 0.029) and 3.31 (95% CI, 1.27 to 8.59; *P* = 0.014; Table 4), respectively. The combination of the two genes revealed a more high HR of 7.25 (95%CI, 2.41 to 21.79; *P* = 0.014). Furthermore, Kaplan-Meier plots revealed that the cumulative hazard of recurrence in methylated and unmethylated VAX1 and LMX1A differed significantly (*P* = 0.034 and *P* = 0.013, respectively). More sigficance was obtained when VAX1 and LMX1A analyzed together (*P*<0.0001, Figure 5). The validation in a small independent cohort consisted of 24 BC and 22 controls revealed the same tendency with HR 9.11(95%CI, 0.89 to 93.7, *P* = 0.063) for the two gene combination with marginal significance owning to the small sample size perhaps (data not shown). These observations underscore the importance for determining the methylation status of VAX1 and LMX1A for the prognosis of disease recurrence.

In additioin to LMX1/VAX1, We also tried to assess the involvement of the other 7 genes in format of two-gene pair combination. The hypermethylation status in some of these genes showed high coincidence with BC recurrence, however this association can not be sustained in the analysis of the follow-up data. Therefore the aberrant methylation in these genes might be the results rather than the trigger of BC recurrence.

The methylation status of ECEL1 and TMEM26 was significantly related to a high degree of tumor differentiation, with HR = 2.43 (95% CI, 1.19 to 4.97; P = 0.01) and 2.32 (95% CI, 1.11 to 4.85; *P* = 0.03), respectively (Figure 4), suggesting that they are involved in BC malignancy and progression.

## Discussion

In this study, we reported the details of establishing biomarkers related to BC methylation, which are as follows: (i) the global methylation profile of both BCCs and BMs by MBD methylCap-seq; (ii) aberrant DNA BC methylation maps through the comparison of the methylation profile of BCCs with BMs; (iii) a panel of promising methylation targets specific to BC; (iv) two informative gene targets informative for BC recurrence; and (v) two methylation genes associated with BC histological differentiation.

Established cancer cell lines are generally expected to share many (if not all) genetic and epigenetic features with tumors *in vivo*, and cancer cell lines are widely used for tumorigenesis studies. A study of human colorectal cancer found that 6 cancer cell lines had methylation patterns that were very similar to the primary tumor with respect to 60 methylation gene targets [29]. Therefore, we began our genome-wide methylation profiling with BCCs and used the clinical sample screening to gather BC-specific methylation information.

Many genes have been reported to be hypermethylated in BC. Recently, studies with new screening approaches, such as methylation arrays, have identified methylation markers with high SN and SP [30], [31], [32]. This genomic screening approach provides an efficient and reliable method for establishing an aberrant methylation profile associated with disease. We adopted the MBD methylCap-seq technique in our studies because it is an open platform for novel methylation loci without prior local sequence knowledge. Therefore, we screened BCCs and compared them to BMs to attempt to discover aberrant BC methylation information.

In addition to the gene promoter methylation status, we obtained the overall genome-wide methylation profile information, which includes different genomic contexts, such as enhancers, downstream regulators, the 5′UTR, exons, introns and microRNAs. The validity of the comparative methylation statuses was confirmed by BSP and MSP. This represents more DNA methylation information for different genomic context features than array methodology, which is limited by probe sequences.

A diagnostic method with high SN and SP is important for any clinically related test. There have been several reports concerning the methylation profiling of urine sediments for BC detection [17], [19], [26], [33], [34], [35], [36]. The techniques used in these studies include conventional MSP, qPCR, nested-MSP, and MethyLight. The diagnostic marker panels are usually composed of 3 to 11 gene targets that range in SN from 77% to 94% and SP from 67% to 100% (Table S6). In contrast, we obtained moderate SN and SP rates of 88.68% and 87.25%, respectively, but these values may be valuable to the Chinese population because there may be disparity between different ethnic populations regarding BC-specific methylation markers [17]. We noted that, except for the wide variation of the diagnostic performance from the different authors, the gene targets were also different. The possible explanations include the different methods and populations used in each of these studies.

To obtain reliable SN and SP values, sufficient numbers for statistic power are necessary. In many of these early studies, small control groups ranging from 6 cases to 20 cases were used [17], [31], [33], [34], [35], [36], which would affect the reliability of the biomarker investigated, particularly the diagnostic SP. Recently, Reiner et al. analyzed 59 control cases [19], and Chung analyzed 110 control cases to correct the problems of small control sample sizes [26] (Table S6). However, in our study, 149 normal controls were analyzed in addition to 212 BC patients. This is perhaps the largest case-control study conducted to analyze urine methylation markers to date. Furthermore, we selected 41 patients with noncancerous urinary lesions to differentiate malignant tumors from benign lesions. To evaluate the possible origin of these targets, their methylation status was investigated in the urine samples from patients before and after the surgical removal of BC. When comparing the diagnostic performance of these methylated targets with cystoscopy, a decent consistency was obtained (an accuracy of 81.25%). All of these procedures were conducted in our study to develop a reliable BC diagnostic panel more objective and less invasive than cystoscopy.

Cancer recurrence presents complex and formidable problems. The detection of BC recurrence primarily depends upon invasive cystoscopy with low compliance, resulting in the need to find a better marker. A new technique for the early prediction of BC recurrence should be able to instantly distinguish the disease risk and provide the proper therapy and necessary intensive vigilance for patients with a high risk of recurrence. This would improve the treatment efficiency and increase the survival interval for high-risk patients while simultaneously reducing unnecessary treatment procedures or vigilance measures, thereby lessening the economic burden on patients at low risk. However, little was previously known regarding the association between DNA methylation and BC recurrence. Tada et al. have found [37] that overexpression of the MDR1 gene may be a prognostic marker for intravesical BC recurrence; however, methylation of its promoter negatively regulates MDR1 expression. Nagraes et al. [38] sought to investigate whether the aberrant DNA methylation of cancer-associated genes was related to urinary BC recurrence and suggested that RARB and RASSF1A gene methylation should be considered potential diagnostic markers, particularly in studies aiming at early recurrence detection. In this study, we found that VAX1 and LMX1A methylation was highly associated with tumor recurrence, suggesting that methylated VAX1 and LMX1A may serve as useful biomarkers to predict BC recurrence.

Currently, the rationality for DNA hypermethylation being a tumor biomarker involves their biological functional relevance in the tumor entity, typically in which DNA hypermethylation in the promoter regions of a gene (tumor suppressor gene, TSG, in most cases) often results in transcription silencing of that gene. Our present study represents the first confirmation for the hypermethylation of 8 genes in bladder cancer, although 4 of them were found to be linked with other tumors before. PROX1 and LMX1 are both hypermethylated with expression silence in breast cancer and gastric cancer [39], [40], representing 2 in line with the presumed epigenetical markers in the present panel; whereas the expression of TAL1 was closely associated with T cell acute lymphoblastic leukemia and hypermethylation of SLC6A20 with malignant mesothelioma, but the exact underlying mechnism is to be elucidated yet [41], [42]. The remaining other 4 genes, VAX1 KCNV1, ECEL1 and TMEM26, were found in the present study to be hypermethylated in BC, but there were no any background record that provided mechanisms in the carcinogenesis and development of any cancer, let alone BC. VAX1 encodes a homeodomain transcription factor known for its role in eye and optic chiasm development [43]; KCNV1 is a gene responsible for a protein related to voltage-gated K(+) channel (Kv) family [44]; ECEL1 encodes a member of the M13 family of endopeptidase [45], while TMEM26 transcripts a protein to be regulated by hedgehog signalling im the mouse limb [46]. Given their hypermethylation status in BC, probabaly these genes also follow the general rules of TSG inactivation in tumor. But the hypothesis needs further studies to prove. At this point, we are making use of the strong association of hypermethylation with BC and focus on the clinical significe it can make.

In using a MBD methylCap-seq procedure, we discovered a series of genes that are frequently methylated in BC. By sequentially screening a series of clinical urine DNA samples, an informative BC biomarker panel was established. This panel is capable of differentiating BC from noncancerous urinary lesions and predicting disease recurrence, which is very important in clinical BC management.

## Supporting Information

Figure S1 **The MSP profile and sequencing verification.** The MSP profile and sequencing verification of the targeted regions of the 8 informative genes in eath of 16 BC samples and 8 normal controls. Both the electrophoretic patterns of the representative MSP data and the sequencing verifcation are shown. P, the positive control with the DNA of the 5637 treated in vitro by M. SssI; N, the negative control (H2O as template). The genomic sequence is aligned with the sequence produced by T-vector cloned with the representative PCR prodcut.
(TIF)

Table S1 **The BSP primer list.**
(XLS)

Table S2 **The MSP primer list.**
(XLS)

Table S3 **The BCC and BM MBD methylCap-seq library characteristics.**
(XLS)

Table S4 **BSP validation of methylation information in BCC and BM libraries.**
(XLS)

Table S5 **The receiver operating characteristics of the informative gene panel for bladder cancer detection.**
(XLS)

Table S6 **The comparison of the performance of the target sets in urine DNA methylation from different authors.**
(XLS)
