# Supplementary figures and images for: Methylcap-Seq Reveals Novel DNA Methylation Markers for the Diagnosis and Recurrence Prediction of Bladder Cancer in a Chinese Population
Source: PLoS One. 2012 Apr 17;7(4):e35175. doi: 10.1371/journal.pone.0035175 (PMC3328468; doi:10.1371/journal.pone.0035175)

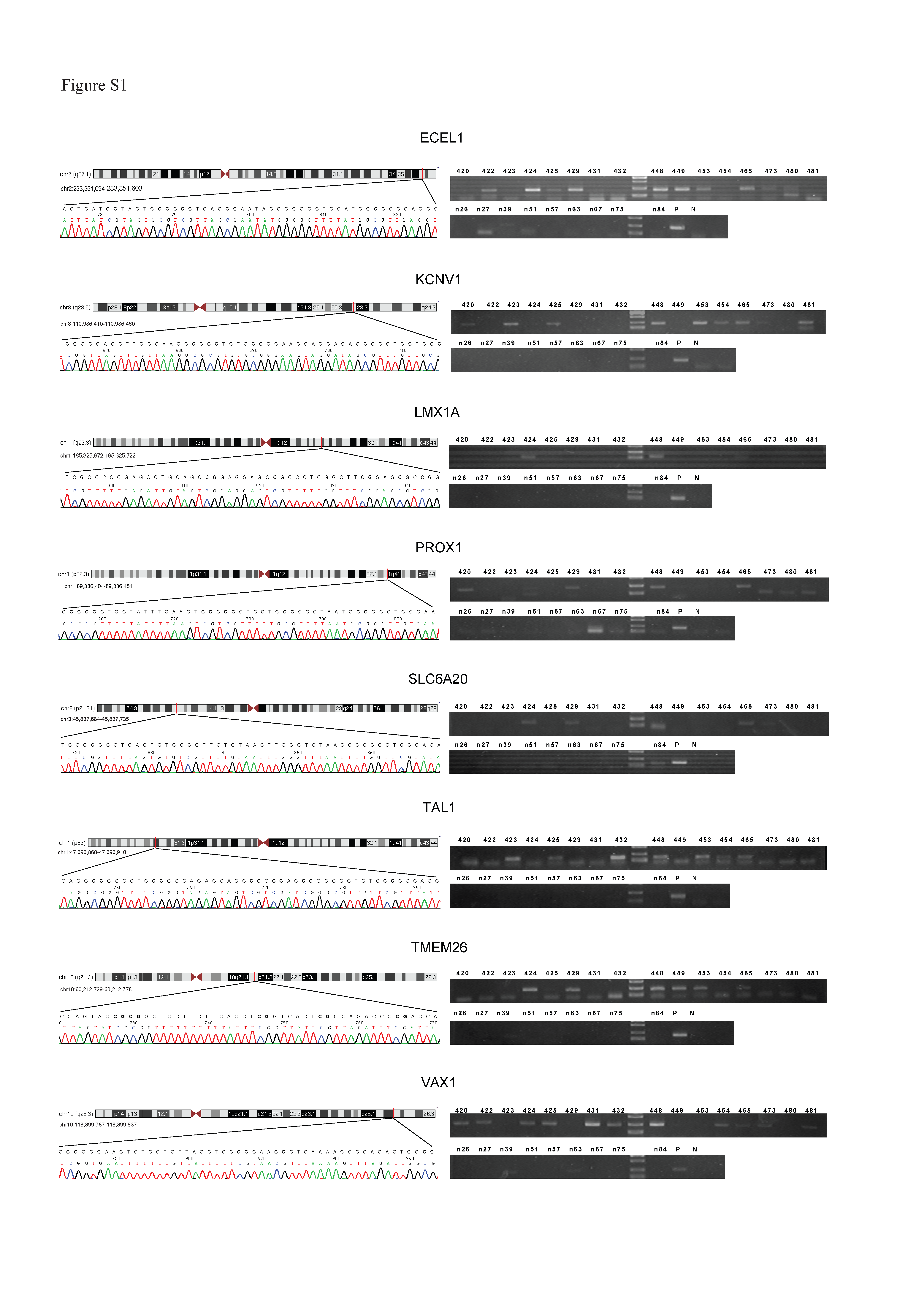

Supplement: Figure S1 — The MSP profile and sequencing verification. The MSP profile and sequencing verification of the targeted regions of the 8 informative genes in eath of 16 BC samples and 8 normal controls. Both the electrophoretic patterns of the representative MSP data and the sequencing verifcation are shown. P, the positive control with the DNA of the 5637 treated in vitro by M. SssI; N, the negative control (H2O as template). The genomic sequence is aligned with the sequence produced by T-vector cloned with the representative PCR prodcut. (TIF) [file pone.0035175.s001.tif]
